# Nonredundant Requirement for Multiple Histone Modifications for the Early Anaphase Release of the Mitotic Exit Regulator Cdc14 from Nucleolar Chromatin

**DOI:** 10.1371/journal.pgen.1000588

**Published:** 2009-08-07

**Authors:** William W. Hwang, Hiten D. Madhani

**Affiliations:** Department of Biochemistry and Biophysics, University of California San Francisco, San Francisco, California, United States of America; European Molecular Biology Laboratory, Germany

## Abstract

In *Saccharomyces cerevisiae*, the conserved phosphatase Cdc14 is required for the exit from mitosis. It is anchored on nucleolar chromatin by the Cfi1/Net1 protein until early anaphase, at which time it is released into the nucleoplasm. Two poorly understood, redundant pathways promote Cdc14 release, the FEAR (Cdc fourteen early release) network and the MEN (mitotic exit network). Through the analysis of genetic interactions, we report here a novel requirement for the ubiquitination of histone H2B by the Bre1 ubiquitin ligase in the cell cycle–dependent release of Cdc14 from nucleolar chromatin when the MEN is inactivated. This function for H2B ubiquitination is mediated by its activation of histone H3 methylation on lysines 4 and 79 (meH3K4 and meH3K79) but, surprisingly, is not dependent on the histone deacetylase (HDAC) Sir2, which associates with Cdc14 on nucleolar chromatin as part of the RENT complex. We also observed a defect in Cdc14 release in cells lacking H3 lysine 36 methylation (meH3K36) and in cells lacking an HDAC recruited by this modification. These histone modifications represent previously unappreciated factors required for the accessibility to and/or action on nucleolar chromatin of FEAR network components. The nonredundant role for these modifications in this context contrasts with the notion of a highly combinatorial code by which histone marks act to control biological processes.

## Author Summary

During proliferation, eukaryotic cells segregate their replicated genome to generate two identical progeny through a highly regulated process called mitosis. Inaccuracy in this process results in cell inviability or aneuploidy. In the *S. cerevisiae* cell cycle, the exit from the mitotic state is triggered by the release of the phosphatase Cdc14 during the anaphase stage of mitosis from nucleolar chromatin, where it is sequestered and kept inactive. The role of chromatin, if any, in the regulation of Cdc14 sequestration and/or release is unexplored. Using genetic analysis, we have discovered that multiple evolutionarily conserved histone modifications are required for the early anaphase release of Cdc14. These include monoubiquitination of histone H2B as well as two methylations of histone H3 on lysines 4 and 79 that require H2B monoubiquitination to occur efficiently. In addition, methylation of H3 on lysine 36 and a histone deacetylase recruited by this modification are also required. We suggest that these histone modifications are required on nucleolar chromatin for the accessibility and/or action of factors involved in the early anaphase release of Cdc14. The nonredundant requirement for multiple chromatin modifications stands in contrast to the popular notion of a highly combinatorial “histone code” for the action of histone modifications.

## Introduction

To ensure the timely and correct inheritance of sister chromatids followed by cytokinesis, eukaryotic cells have evolved sophisticated regulatory networks. Part of the regulatory complexity involves the requirement that a variety of soluble proteins communicate with tightly chromatin-bound factors on chromosomes. An example of such a mechanism in the budding yeast *S. cerevisiae* is the control of Cdc14, a conserved and essential protein phosphatase. Outside of anaphase, Cdc14 is kept away from its soluble substrates by being tightly associated with nucleolar chromatin through its inhibitor, the nucleolar protein Cfi1/Net1. Upon entry into anaphase, however, this interaction is dissolved via the phosphorylation of Cfi/Net1, which is necessary for the release of Cdc14 from the nucleolus [1]–[3]. Liberated Cdc14 dephosphorylates mitotic Clb cyclin dependent kinases (Clb-CDKs) and their substrates to promote exit from mitosis [3]–[6].

Work in the last decade has elucidated two pathways, dubbed the FEAR (Cdc Fourteen Early Anaphase Release) network and the MEN (Mitotic Exit Network), that together control the release of Cdc14 [7],[8]. The MEN, the first and better characterized of the two networks, is a signaling cascade which is controlled by the GTPase Tem1 and its upstream regulators, the GTPase activating complex Bub2-Bfa1 and the GTP exchange factor Lte1. Before and during early anaphase, Tem1 is kept in its inactive GDP state by association with Bub2-Bfa1 at the spindle pole body (SPB). As the SPB enters the daughter cell during mid to late anaphase, Tem1 is relieved of inhibition by exposure to bud localized Lte1 and activates a kinase cascade consisting of Cdc15 and the Dbf2/Mob1 complex [9]–[14]. Finally, through an uncharacterized mechanism, Dbf2/Mob1 directs the release of Cdc14, possibly through direct phosphorylation of Cfi1/Net1 [14].

**Figure 1:**
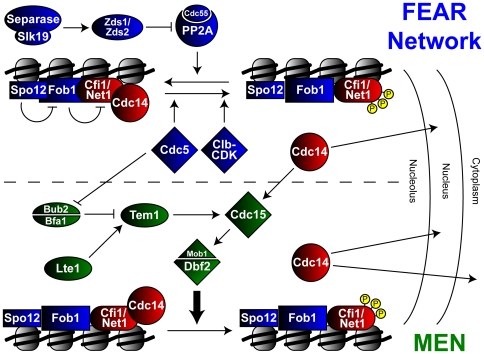
Regulation of Cdc14 release by the MEN and FEAR network. See Introduction for description.

MEN mutants, however, still display a transient release of Cdc14 early in anaphase [15]–[18]. This observation led to the identification of the FEAR network, encoded by a set of genes that when mutated in combination with mutations in the MEN are unable to release Cdc14 from the nucleolus [16]. Genetic epistasis experiments separate FEAR network components into two pathways [19]. One is regulated by a non-proteolytic function of Esp1, the cohesin protease also known as separase, which acts in concert with the kinetochore associated protein Slk19 to inhibit the paralogs Zds1 and Zds2 [16],[18],[20]. These latter proteins appear to inhibit a type 2A protein phosphatase called PP2A^Cdc55^ that otherwise reverses the phosphorylation of Cfi1/Net1 by Clb-CDK [20]. The anaphase inactivation of PP2A^Cdc55^ has been suggested to lead to the accumulation of phosphorylated, inactive Cfi1/Net1 and consequent release of Cdc14 [21]. The second branch of the FEAR network includes the nucleolar protein Spo12, its homolog Bns1, and Fob1, which binds Spo12 to relieve its inhibitory interaction with Cfi1/Net1 [16],[19],[22],[23]. The initial burst of Cdc14 release promoted by the FEAR network feeds forward to activate the MEN, thus promoting its continued release until the end of mitosis [13],[16],[17],[24],[25]. Bridging the two pathways is the Polo-like kinase Cdc5, which can regulate the MEN by inhibition of the Bub2/Bfa1 complex but is also required for FEAR network mediated Cdc14 release downstream of or parallel to separase by potential direct phosphorylation of Net1 [1],[16],[19],[26],[27] (Figure 1). The biochemical mechanisms by which FEAR network components combine to regulate early anaphase release of Cdc14 remain a mystery.

Cfi1/Net1, in addition to regulating Cdc14 function, is localized onto rDNA chromatin with the histone deacetylase Sir2 in a protein complex named RENT (regulator of nucleolar silencing and telophase exit) to regulate the silencing of rDNA heterochromatin, recombination of the rDNA repeats, and RNA Polymerase I transcription [5], [6], [28]–[30]. The localization of RENT to the NTS1 (non-transcribed spacer) region of rDNA is dependent on Fob1, which also has multiple functions at this region including blocking replication fork progression to prevent the collision of convergent DNA polymerases [30]. Curiously, Cdc14 is sequestered on rDNA chromatin specifically at this RFB (replication fork block) along with the other members of the RENT complex and Fob1, leading to speculation on whether or not the regulation for these other activities at rDNA overlap with the regulation of Cdc14 release [23],[30].

Recent studies have revealed a role for histone modifications in regulating rDNA functions. In particular, the methylation of histone H3 on Lysine 4 and 79 by the methyltransferases Set1 and Dot1 respectively are required for robust rDNA silencing [31]–[35]. These modifications require the presence of monoubiquitination on Lysine 123 of histone H2B (ubH2B-K123), mediated by the E2 ubiquitin conjugating enzyme Rad6 and the E3 ubiquitin ligase Bre1 [36]–[38]. Deletion of the H2B deubiquitinating enzyme Ubp10 results in decreased Sir2 association at rDNA and increased the recombination frequency of rDNA repeats [39], suggesting that histone modifications can play a direct role in regulating rDNA functions.

Based on recent genetic evidence from large-scale synthetic lethal screens linking the histone ubiquitination machinery to members of the MEN [40],[41], we investigated the possibility that histone modifications may be required for efficient exit from mitosis. We verified the genetic interactions cited above and demonstrate a requirement for histone ubiquitination in the early nucleolar release of Cdc14 during early anaphase in the absence of MEN activity. This requirement is dependent on the activation of histone methylation by ubH2B-K123 but is independent of Sir2. Finally, we show that these effects are not due to aberrant regulation of known FEAR network components and can be extended to other histone modifications.

## Results

### H2B-K123 Ubiquitination Is Required for Cell Viability and Full Release of Cdc14 from the Nucleolus When MEN Is Inactive

**Figure 2:**
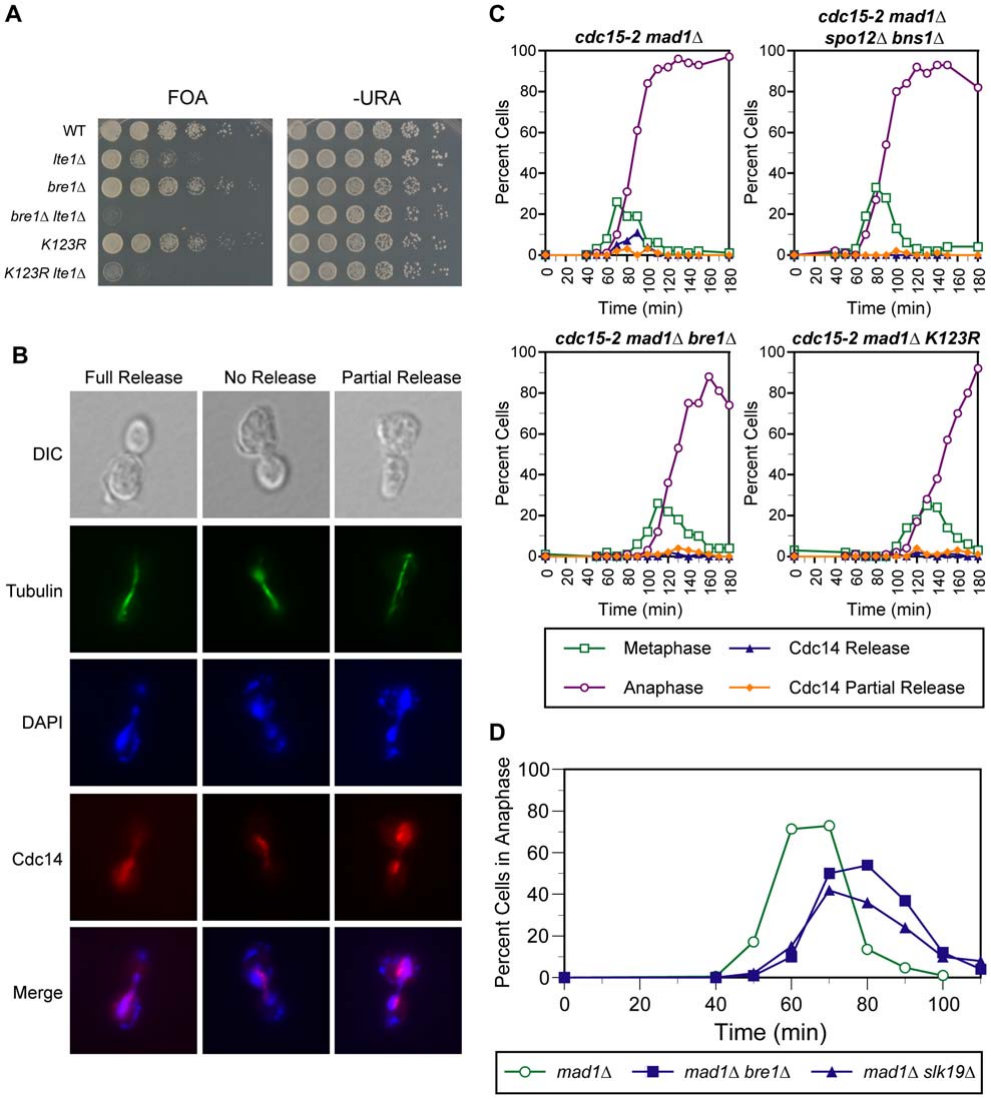
Mutants unable to ubiquitinate histone H2B-K123 display growth and Cdc14 release defects when combined with MEN mutants. (A) Genetic interaction of the *bre1Δ* and *htb1-K123R htb2-K123R* mutations with the *lte1Δ* mutation. Shown is the growth on 5-FOA and SD –Ura minimal media of five-fold serial dilutions of strains of the indicated genotype that initially harbored an *URA3*-marked centromeric plasmid encoding *LTE1*. Plates were incubated for 3 days at 30°C. See Materials and Methods for details. (B) Representative images from Cdc14 release assay. Cells are assayed for spindle morphology and Cdc14 release phenotype by indirect immunofluorescence with anti-tubulin or anti-HA, which recognizes the HA-tagged version of Cdc14 in all strains. Cells are visualized by nuclear staining with 4′,6-diamidino-2-phenylindole (DAPI) and by differential interference contrast (DIC) optics for comparison. No release refers to dense nucleolar staining of Cdc14-HA, full release refers to cells with uniform Cdc14-HA localization throughout the nucleus, while partial release refers to cells with both nuclear and residual nucleolar staining. (C) Time course analysis. The temporal profile of Cdc14 release from nucleolar sequestration is displayed for *cdc15-2 mad1Δ* mutants harboring either no additional mutation, *bre1Δ*, *htb1-K123R htb2-K123R*, or *spo12Δ bns1Δ* mutations. Alpha-factor arrested cells are released into YPD media at 37°C to inactivate the *cdc15-2* mutant gene product and timepoints are processed for indirect immunofluorescence as described in Figure 2B. Approximately 200 cells were scored for each timepoint for metaphase or anaphase spindles and for full or partial release of Cdc14-HA. (D) Time course analysis. Cell cycle progression of *mad1Δ*, *mad1Δ bre1Δ*, and *mad1Δ slk19Δ* mutants arrested and released from alpha-factor arrest was measured by scoring ∼200 cells from each timepoint for anaphase spindles by indirect immunofluorescence.

We reasoned that new functions for Bre1, the conserved ubiquitin ligase for ubH2B-K123, might be illuminated through the identification of mutants that display synthetic growth defects when combined with *bre1Δ* mutants. High-throughput microarray-based studies reported a genetic interaction between *bre1Δ* and deletion of the GTP exchange factor Lte1, a regulator of the MEN [40],[41]. Such a genetic interaction is characteristic for members of the FEAR network, which act in a partially redundant manner with the MEN to regulate the essential release of Cdc14 from the nucleolus and promote exit from mitosis (Figure 1). Specifically, defects in the MEN render FEAR network components essential for viability and is a defining characteristic of FEAR members [16]. Therefore, we investigated the possible requirement for Bre1 in the release of Cdc14 in early anaphase. We confirmed the reported synthetic interaction and observed the inviability of the *bre1Δ lte1Δ* double mutant as determined by its inability to lose a *URA3*-marked plasmid encoding *LTE1* as assayed by lack of growth on 5-FOA media (Figure 2A). The lethal phenotype of the *bre1Δ lte1Δ* mutant was rescued by the deletion of *BUB2*, an upstream negative regulator of the MEN, consistent with the notion that lethality of the *bre1Δ lte1Δ* mutant results from a defect in mitotic exit (Figure S1). Synthetic lethality was also observed in double mutants containing a deletion of *LTE1* with either *RAD6*, which encodes the E2 enzyme for H2B ubiquitination, or *LGE1*, which encodes a Bre1-interacting protein that is required for ubH2B-K123 (Figure S1).

Since histone H2B is the only known substrate for Bre1, we hypothesized that ubH2B-K123 mediates the mitotic exit functions for Bre1. To test this hypothesis, we generated a strain replacing the codon for lysine 123 of both histone H2B genes with one coding for arginine (*htb1-K123R htb2-K123R*), rendering histone H2B unable to be ubiquitinated. We first examined the phenotype of a triple mutant with *lte1Δ*. The *htb1-K123R htb2-K123R lte1Δ* triple mutant displayed a very severe growth defect (Figure 2A). This result points to a role for ubH2B-K123 as the downstream effector of Bre1 in promoting mitotic exit. However, the ability to obtain viable, albeit very slow, colony growth for the triple mutant compared to the inviability of the *bre1Δ lte1Δ* mutant is consistent with an additional function for Bre1 that is independent of H2B ubiquitination.

To test more directly the potential roles of Bre1 and ubH2B-K123 in FEAR network function, we took advantage of the observation that cells synchronized for cell cycle progression (by arrest in G1 with mating pheromone followed by release into the cell cycle) transiently release Cdc14 (as determined by indirect immunofluorescence) during early anaphase when the MEN was inactivated by the *cdc15-2* mutation. In this MEN mutant background, Slk19 and Spo12 have been reported to be required for full release of Cdc14 [16]. In our experiments, *cdc15-2 mad1Δ slk19Δ* mutants (*MAD1* is deleted to allow comparison to *slk19Δ* mutants, which exhibit spindle checkpoint defects [42]) displayed a complete defect in Cdc14 release during anaphase, while *cdc15-2 mad1Δ spo12Δ bns1Δ* mutants displayed a small population with a partial release phenotype, characterized by Cdc14 release combined with residual sequestration in the nucleolus (Figure 2B and 2C). This is consistent with the published phenotypes of FEAR mutants [16]. When *cdc15-2 mad1Δ bre1Δ* mutants were assayed, they showed a defect in Cdc14 release during early anaphase reminiscent of *cdc15-2 mad1Δ spo12Δ bns1Δ* mutants. Similarly, *cdc15-2 mad1Δ htb1-K123R htb2-K123R* quadruple mutants were also unable to fully release Cdc14 (Figure 2C).

To ensure that the Cdc14 release phenotypes we observed were not the result of asynchronous release from arrest resulting in the imprecise measurement of anaphase, we assayed for Cdc14 release phenotypes in individual cells while measuring spindle lengths as a more precise marker of anaphase progression. We observe full release of Cdc14 in a high percentage of *cdc15-2 mad1Δ* cells with short spindles (2–6 µm), while *cdc15-2 mad1Δ bre1Δ* mutants only display a small percentage of Cdc14 that is partially released, in accordance with our timecourse results (Figure S2). In addition, we observed that *cdc15-2 mad1Δ bre1Δ* and *cdc15-2 mad1Δ htb1-K123R htb2-K123R* mutants displayed a ∼40 minute delay in progression into metaphase and anaphase. This defect in *cdc15-2 mad1Δ bre1Δ* mutants was a result of a prior cell cycle delay of ∼30–40 minutes that occurs prior to G2, as demonstrated by the analysis of DNA content and most likely does not reflect a defect in mitotic entry (Figure S3).

Another defining characteristic of FEAR network mutants is that they are delayed in cell cycle progression due to the elongation of anaphase [16]. To test whether or not *bre1Δ* mutants display this defect, we measured the progression through the cell cycle of synchronized *mad1Δ*, *mad1Δ bre1Δ*, and *mad1Δ slk19Δ* mutant cells. *mad1Δ bre1Δ* mutants, like *mad1Δ slk19Δ* mutants, exhibited a 10 minute increase in time spent in anaphase when compared to *mad1Δ* mutants (∼50 minutes total time in anaphase for *mad1Δ* mutants vs. ∼60 minutes for *mad1Δ bre1Δ* or *mad1Δ slk19Δ* mutants) (Figure 2D).

### Cdc14 Release Defects Observed in *bre1Δ* Cannot Be Explained by Alterations in Expression and Localization of FEAR Network Members

**Figure 3:**
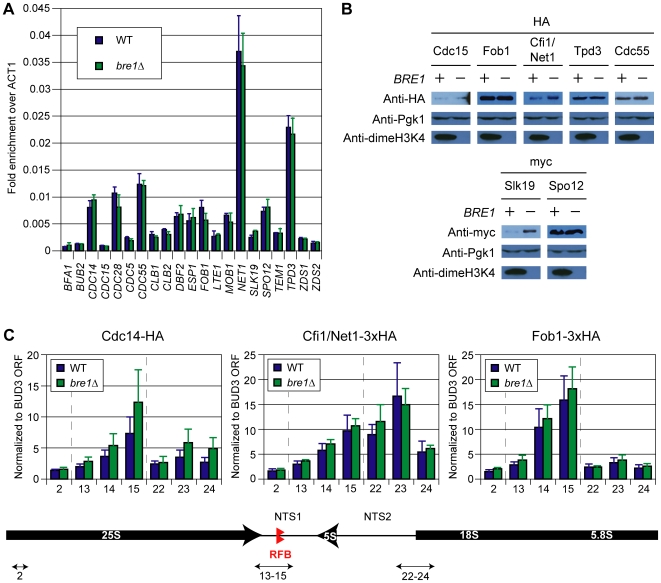
Changes in expression and chromatin association of mitotic exit regulators do not explain Cdc14 release defects in *bre1Δ* mutants. (A) Reverse transcription coupled to quantitative PCR analysis (RT–QPCR) of known mitotic exit regulators. Levels of cDNA generated from RT reactions of wildtype and *bre1Δ* total RNA were measured by QPCR. The mean values and standard deviations presented represent triplicate samples for each strain and are normalized to *ACT1* levels. Reactions were performed without RT (−RT) to establish background DNA contamination. *BFA1*, the lowest level transcript, was enriched 15 fold over the −RT control (data not shown). (B) Western blots of epitope-tagged regulators of Cdc14 release. Cdc15, Fob1, Cfi1/Net1, Tpd3 and Cdc55 were tagged with 3xHA while Slk19 and Spo12 were tagged with 4xmyc and 13xmyc, respectively. The blots were cut into two parts to simultaneously probe for the epitope tag on the upper part and with anti-dimeH3K4 on the lower to demonstrate loss of *BRE1*. The upper blots were stripped and reprobed with anti-Pgk1 to control for loading. (C) Chromatin immunoprecipitation of HA tagged Cdc14, 3xHA tagged Cfi1/Net1, and 3xHA tagged Fob1 in wild type and bre1Δ mutant backgrounds. The numbers on the x-axis refer to primer pairs used to amplify the immunoprecipitated samples corresponding to locations at the rDNA locus as depicted in the schematic below and from [62]. Quantitative PCR mean values and standard deviations presented represent triplicate samples for each rDNA primer set and are normalized to values from a control primer set located within the open reading frame (ORF) of *BUD3*. Dotted lines separate the primer pairs corresponding to different regions of the rDNA locus. Bottom panel: NTS 1 and 2 = non-transcribed spacer regions 1 and 2. RFB = replication fork block. *5S*, *5.8S*, *18S*, *25S* = rDNA open reading frames.

Several studies, including our own, have established transcriptional changes in the *bre1Δ* mutant and mutants in H2B ubiquitination (our unpublished observations), [43]. It is possible that the defects we observe for Cdc14 release in *bre1Δ* are due to transcriptional changes of genes encoding for known regulators of mitotic exit. To address this possibility, we measured the mRNA levels of known MEN and FEAR network regulators by reverse transcription coupled to quantitative PCR analysis (RT-QPCR). We observed no significant changes in transcript levels between wildtype and *bre1Δ* mutants (Figure 3A). In addition, we epitope-tagged several key FEAR network components and compared their protein expression levels in wild type cells to *bre1Δ* mutants to account for any post-transcriptional regulation of these proteins (Figure 3B). Levels of the Cdc14 release activators Slk19, Spo12, and Cdc15 would be expected to decrease in *bre1Δ* mutants if expression changes were the cause of the mitotic exit defects observed for histone H2B ubiquitination mutants. We observed no decreases in these activators; in fact, we found that the levels of Slk19 are increased in *bre1Δ* cells (Figure 3B). Conversely, inhibitors of Cdc14 release such as Fob1, Cfi/Net1, and the PP2A regulatory subunits Cdc55 and Tpd3, would be increased in expression to explain the Cdc14 release defects in *bre1Δ* mutants. We observed no changes in *bre1Δ* mutants for the inhibitors listed above except Cfi1/Net1, which displays an increase in expression (Figure 3B).

This increase in Cfi1/Net1 levels provided a possible explanation for the mitotic exit defects observed for *bre1Δ* mutants that we sought to investigate further. Cdc14 sequestration in nucleolar chromatin is dependent on two proteins, Fob1 and Cfi1/Net1, which interact biochemically and co-localize to the NTS1 region of rDNA along with Cdc14 [23],[30]. If the increase in total Cfi1/Net1 levels corresponds to an increase in Cfi1/Net1 occupancy at NTS1 in *bre1Δ* mutants, Cdc14 sequestration could be increased, causing the observed Cdc14 release defects. Furthermore, even though total Fob1 levels are unchanged in *bre1Δ* mutants, Fob1 bound to chromatin could in principle be increased by a loss of uH2B-K123, leading to defects in mitotic exit similar to those observed previously for strains overexpressing Fob1 [23]. To test these possibilities, the quantitative association of epitope-tagged Cfi1/Net1 and Fob1 to the rDNA loci was measured by chromatin immunoprecipitation (ChIP) in wild type and *bre1Δ* strains. We observed enrichment of Cfi1/Net1 and Fob1 at the NTS1 region of rDNA, consistent with previous reports, but did not detect any significant changes in *bre1Δ* mutants (Figure 3B). Thus, the mitotic exit defects of *bre1*Δ mutants cannot be explained by detectable changes in the localization profiles of known chromatin-associated Cdc14 regulators. Finally, it is possible that the excess Cfi1/Net1 observed in *bre1Δ* cells could be aberrantly sequestering Cdc14 elsewhere in the nucleolus, rendering Cdc14 inaccessible to activation by the FEAR network. To address this possibility, we measured Cdc14 levels at rDNA by ChIP. We observed no significant alteration of the Cdc14 signal at these endogenous sites of sequestration.

### Cdc14 Release Defects of Cells Defective for ubH2B-K123 Can Be Explained by Defects in Downstream Methylation of Histone H3

**Figure 4:**
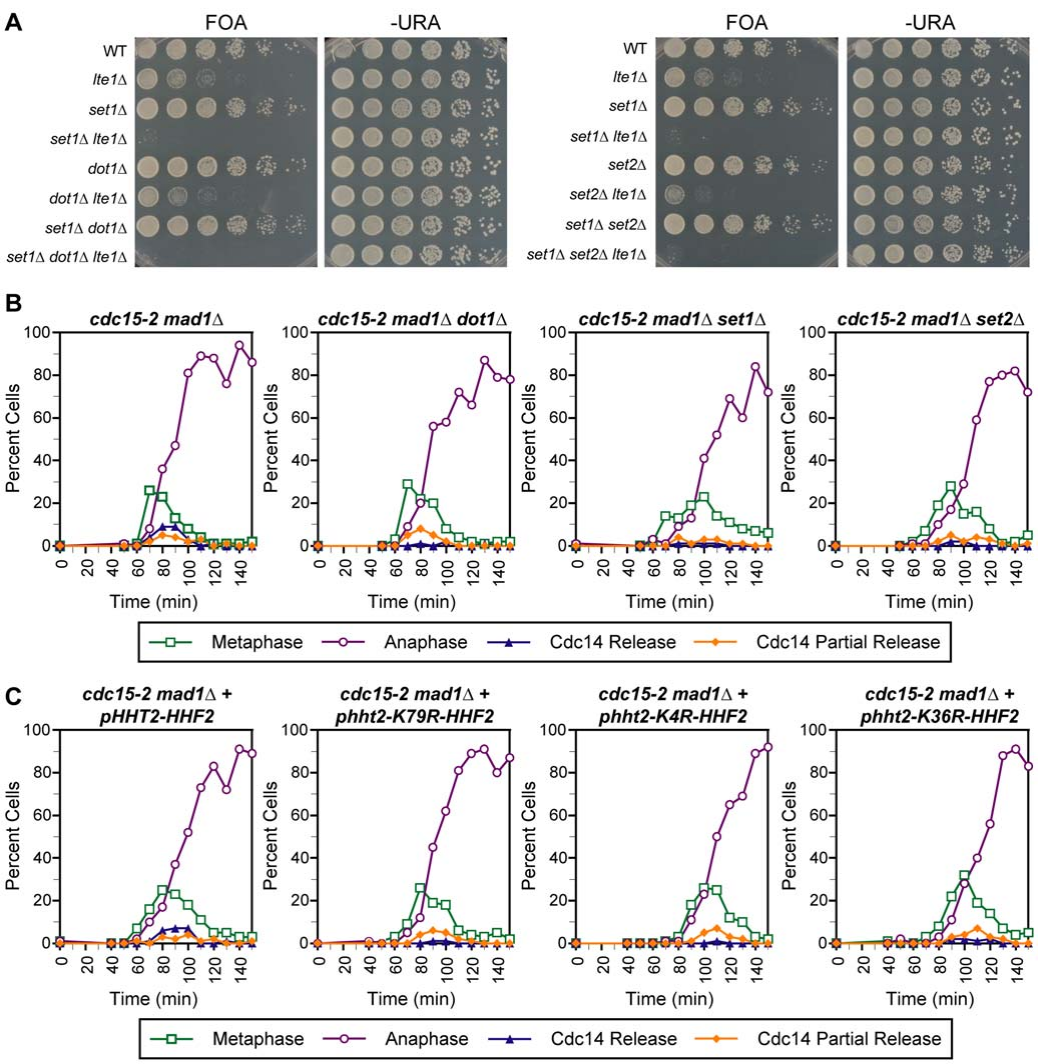
Genetic interaction and Cdc14 release profile of histone H3 methylase mutants combined with MEN mutants. (A) Genetic interactions of *set1Δ*, *dot1Δ*, *set2Δ*, *set1Δ dot1Δ*, and *set1Δ set2Δ* mutations with the *lte1Δ* mutation. Strains were assayed as in Figure 2A. (B,C) The temporal profile of Cdc14 release from nucleolar sequestration is displayed for strains of the indicated genotypes. Time courses were performed as described in Figure 2C.

To date, the only characterized role for H2B ubiquitination is to promote the downstream methylation of H3K4 and H3K79. Therefore we tested the hypothesis that the Cdc14 release defects we observed in *bre1Δ* and *htb1-K123R htb2-K123R* strains were due to the absence of meH3K4 and/or meH3K79. We first assayed the phenotype of double mutants of *lte1Δ* and deletions of Set1 and Dot1, the methyltransferases responsible for meH3K4 and meH3K79, respectively. The *lte1Δ set1Δ* mutant displayed a synthetic lethal phenotype while the *lte1Δ dot1Δ* mutant exhibited a similar growth defect to the *lte1Δ* single mutant (Figure 4A). This phenotype for *set1Δ* is unaffected by the loss of Dot1, as the *set1Δ dot1Δ lte1Δ* triple mutant is also inviable (Figure 4A). This genetic data suggested that the requirement for H2B ubiquitination in mitotic exit might be specific to its downstream activation of meH3K4.

To test this prediction directly, we assayed for the early anaphase Cdc14 release in *cdc15-2 mad1Δ* strains containing deletions of *SET1* and *DOT1* (Figure 4B). The *cdc15-2 mad1Δ set1Δ* mutant was unable to release Cdc14, consistent with the genetic interaction between *set1Δ* and *lte1Δ* and similar to the phenotype we observed for *bre1Δ* and *htb1-K123R htb2-K123R* mutants. However, in contrast the predictions from the genetic interactions, *cdc15-2 mad1Δ dot1Δ* mutants are also unable to fully release Cdc14, though the release defect is milder than that observed for *set1Δ* mutants. This discrepancy may be explained by differing thresholds for function in the two assays and/or the fact that eliminating the function of Cdc15, the central and essential kinase of the MEN, is a more severe perturbation of mitotic exit than the deletion of the non-essential upstream regulator Lte1. In addition, the defect in Cdc14 release of the methylase mutants is due to their primary function in histone methylation, as unmethylatable lysine to arginine mutants of H3K4 and H3K79 (H3K4R and H3K79R) were also unable to efficiently release Cdc14 (Figure 4C). We note that *set1Δ* mutants exhibited a more severe Cdc14 release defect compared to the H3K4R mutant. This is potentially explained by the loss, in *set1Δ* mutants, of the Set1-dependent methylation of the kinetochore protein Dam1 or other unknown targets [44]. Taken together, the genetic and Cdc14 release data suggests that the loss of methylation on H3K4 and H3K79 are required for efficient release of Cdc14 in the absence of MEN, suggesting a more generalized requirement for histone modifications in this process.

### Cdc14 Release in the Absence of MEN Activity Requires the Methylation of meH3K36 and Its Recruited Histone Deacetylase Rpd3

Our data suggest that in the absence of MEN activity, multiple histone H3 methylation events are required for the complete release of Cdc14 from sequestration. To address the specificity of this requirement, we assayed the phenotype of double mutants of *lte1Δ* and deletion of Set2, the remaining histone H3 methylase in yeast, which catalyzes the modification of lysine 36. Surprisingly, the *lte1Δ set2Δ* double mutant displayed a slight synthetic sick phenotype, suggesting a potential role for meH3K36 in promoting mitotic exit (Figure 4A). This was confirmed by examination of the Cdc14 release profile of a *cdc15-2 mad1Δ* strain carrying a deletion of Set2 or an unmethylatable lysine to arginine mutation of H3K36 (H3K36R). These mutants also displayed a defect in Cdc14 release that was similar to the defect observed in mutants unable to methylate H3K4 and H3K79 (Figure 4B and 4C).

**Figure 5:**
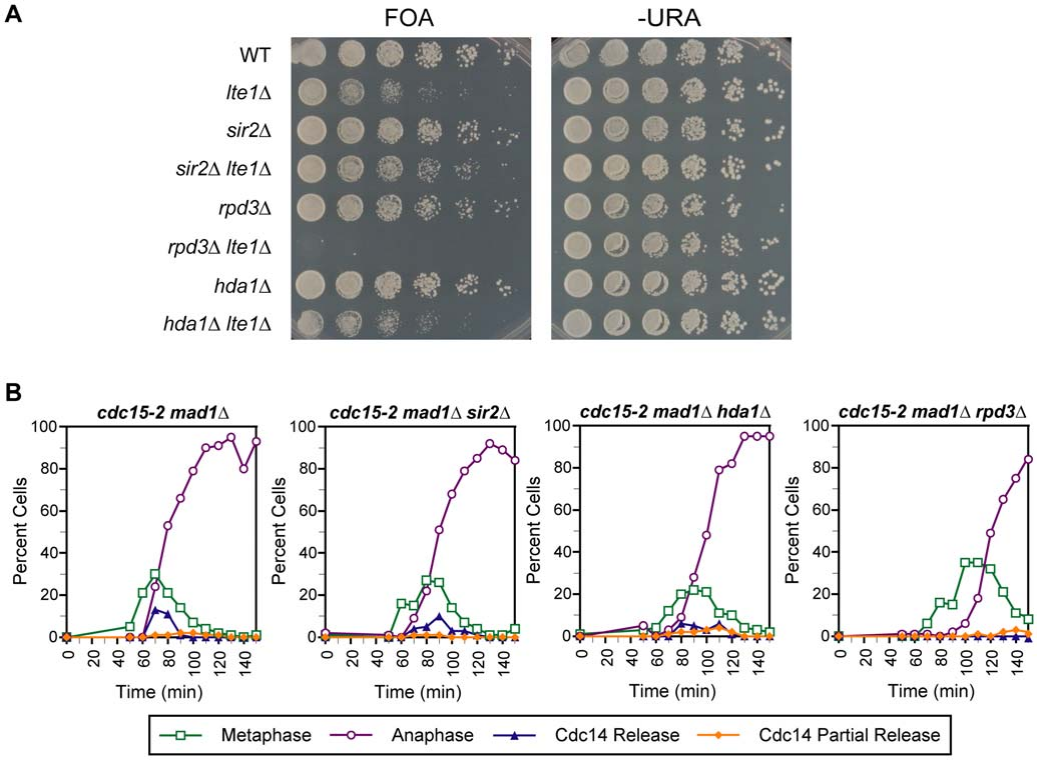
Genetic interaction and Cdc14 release profile of histone deacetylase mutants combined with MEN mutants. (A) Genetic interactions of the *sir2Δ*, *hda1Δ*, and *rpd3Δ* mutations with the *lte1Δ* mutation. Strains were assayed as in Figure 2A. (B) Time course analysis. The temporal profile of Cdc14 release from nucleolar sequestration is displayed for strains of the indicated genotypes. Time courses were performed as described in Figure 2C.

The requirement for meH3K36 for efficient Cdc14 release is intriguing because this methylation has been demonstrated to recruit the Rpd3 histone deacetylase complex. Large-scale screens have detected a synthetic lethal interaction between *lte1Δ* and *rpd3Δ* mutants [40], which we reconfirmed using our plasmid shuffle strategy (Figure 5A) raising the possibility that histone deacetylation by Rpd3 is an additional requirement for Cdc14 release. To address this possibility, we assayed for Cdc14 release in a *cdc15-2 mad1Δ rpd3Δ* mutant and observed a complete absence of release, suggesting that histone deacetylation is necessary for Cdc14 release (Figure 5B). However, this requirement appears to be specific to deacetylation by Rpd3, as deletion of Hda1, a related histone deacetylase that displays overlapping functions with Rpd3 [45], did not show genetic interactions with *lte1Δ* and displays only a very slight Cdc14 release defect (Figure 5A and 5B). Similarly, a mutant strain deleted for Sir2, the histone deacetylase responsible for rDNA silencing in yeast, displayed no synthetic growth defect when combined with *lte1Δ* and was still able to release Cdc14 in the absence of MEN activity (Figure 5A and 5B).

### Cdc14 Release Defects of FEAR Pathway and Histone Modification Mutants Do Not Require the Histone Deacetylase Sir2

**Figure 6:**
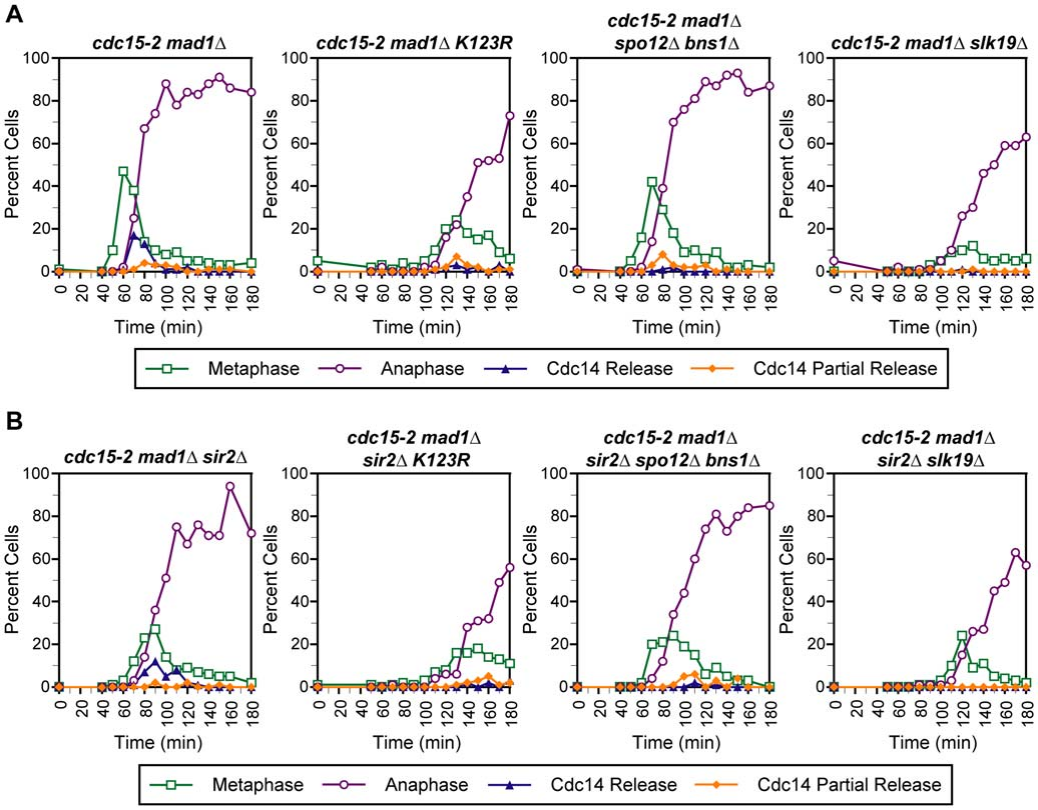
Sir2 is not responsible for Cdc14 release defects of *htb1-K123R htb2-K123R* and FEAR network mutants. (A,B) Time course analysis. The temporal profile of Cdc14 release from nucleolar sequestration is displayed for strains of the indicated genotypes. Time courses were performed as described in Figure 2C.

The data above indicate that the loss of Sir2 does not affect the ability of cells to release Cdc14 in the absence of MEN. However, recent studies from our laboratory and others have shown that histone H3 methylation can antagonize the association and function of Sir2 [46]. In addition, cells lacking Ubp10, an H2B-K123 deubiquitinating enzyme, display a decrease in Sir2 binding at the NTS1 region of rDNA where Cdc14 is bound [39]. This raised the possibility that the Cdc14 release defects that we observe in histone modification mutants result from increased or altered localization of Sir2 at rDNA. To address this possibility, we assayed a *cdc15-2 mad1Δ htb1-K123R htb2-K123R sir2Δ* mutant to determine whether or not deletion of Sir2 could rescue the Cdc14 release defect of *cdc15-2 mad1Δ htb1-K123R htb2-K123R* cells. No changes in Cdc14 release were observed when the two mutants were compared (Figure 6A and 6B), suggesting that altered Sir2 function is not responsible for the Cdc14 release phenotype of *htb1-K123R htb2-K123R* mutants. Furthermore, deletion of Sir2 did not rescue the Cdc14 release defects of *spo12Δ bns1Δ* or *slk19Δ* mutants, demonstrating that aberrant Sir2 function is not the cause of the Cdc14 release defects observed in known FEAR pathway mutants (Figure 6A and 6B).

## Discussion

Although typically thought of as a site for transcription of tandem arrays of rDNA genes, rRNA processing, and ribosome assembly, the nucleolus plays other critical roles in cellular regulation. The anchoring of the conserved eukaryotic phosphatase Cdc14 in the nucleolus is a prominent example of such a function. Despite the physical association of Cdc14 and its sequestration machinery with nucleolar chromatin, the impact of chromatin modifications on this mechanism has not been reported previously.

In this study, we provide evidence for a role for the conserved ubiquitin ligase Bre1 and monoubiquitination of histone H2B in the early phase of mitotic exit. First, in cells lacking the MEN component Lte1, we observed that deletion of *BRE1* or mutation of the codon encoding the target lysine in both H2B genes results in cellular inviability or severe growth defects, respectively. Such a phenotype is characteristic of mutants in components of the FEAR network responsible for the early anaphase release of Cdc14. Second, we found that Bre1 and ubH2B-K123 are indeed required for full Cdc14 release during early anaphase when the MEN is inactivated by the *cdc15-2* mutant.

Furthermore, we established that this requirement for ubH2B-K123 in Cdc14 release is dependent on the downstream effects of ubH2B-K123 on the methylation of H3K4 and H3K79, as strains unable to be methylated on either residue are also defective for early anaphase Cdc14 release. We also observed Cdc14 release defects for a mutant unable to be methylated by Set2 on H3K36, which was surprising since H3K36 methylation does not require ubH2B-K123 [47]. Nonetheless, these data demonstrate that all three of the known methylation marks on histone H3 in *S. cerevisiae* play a role in this process. These data may also help to explain the observation that a haploid H3K4R,36R,79R strain mutated for all three H3 methylation sites displays an increased population of cells with 2C DNA content [48].

**Figure 7:**
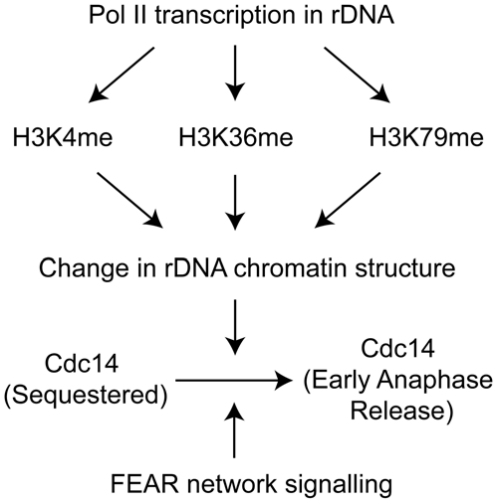
Nonredundant roles for histone modifications in promoting early anaphase release of Cdc14. This model proposes that the three known histone methylations present in *S. cerevisiae* function nonredundantly to change nucleolar chromatin structure and permit FEAR pathway signaling.

The requirement for histone modifications in mitotic exit is most simply explained by the direct modification of nucleolar chromatin (Figure 7). We demonstrated that the Cdc14 release defects we observe are not correlated with changes in expression levels of known Cdc14 release factors, as the transcript and protein levels of regulators are unchanged or increased in *bre1Δ* mutants. The sole exception is the increase in levels of Cfi1/Net1, which could potentially cause an increase in Cdc14 sequestration. However, this increase does not change the levels of Cfi1/Net1 or Fob1 associated with chromatin at the rDNA locus as measured by ChIP. The presence of both H3K4 and K79 methylation has been reported at rDNA chromatin by ChIP, consistent with a direct role for these modifications in affecting rDNA function [34],[35],[49],[50]. Dot1, the H3K79 methylase, has also been reported to be present in the nucleolus by microscopy of cycling cells and spread mitotic nuclei [51]. Lastly, deletion of Ubp10, a deubiquitinating enzyme for ubH2B-K123, increases the levels of both meH3K4 and K79 at the NTS1 region, suggesting that ubH2B-K123 is also present at the site of Cdc14 sequestration and is able to promote the methylation of H3K4 and K79 at this locus [39]. These modifications may depend on the transcription of this region by RNA polymerase II (Pol II), since there is a reported transcript produced from a promoter in the NTS1 region and Pol II has been detected in this region by ChIP [50],[52],[53].

In addition to showing that histone H2B ubiquitination and histone H3 methylation are required for efficient Cdc14 release, we verified the reported genetic interaction of *rpd3Δ* and *lte1Δ* [40] and demonstrated defects in Cdc14 release of *rpd3Δ* mutants when MEN activity was inhibited. Unlike histone methylation, however, the requirement for histone deacetylation in Cdc14 release was specific to deacetylation by Rpd3 and not shared by other HDACs such as Hda1 and Sir2. These results are in agreement with expression microarray data showing no significant overlap between *sir2Δ* and *rpd3Δ* mutant expression profiles and the overrepresentation of cell cycle related transcripts in *rpd3Δ* that is not observed in *hda1Δ* mutants [45]. The transcriptional changes of cell cycle genes in *rpd3Δ* mutants suggest a possible indirect transcriptional mechanism for the requirement for Rpd3 in Cdc14 release but given the many reports linking Rpd3 to rDNA functions a direct role at rDNA cannot be excluded [54],[55].

Although our studies point to a role for histone modifications in controlling the early anaphase release of Cdc14, understanding the underlying biochemical mechanisms involved will require further studies. An appealing possibility is that histone modifications may serve to maintain a permissive state for mitotic exit regulators to access the chromatin associated factors Cfi1/Net1 and Fob1 at the onset of anaphase and to enact the release of Cdc14 (Figure 7). Surprisingly, our genetic experiments indicate that this requirement is independent of the action of the Cdc14-associated histone deacetylase Sir2 and removal of Sir2 did not bypass the requirement for histone modifications in Cdc14 release. Potential candidates whose access to Cfi1/Net1 could be affected by this alteration of chromatin function include Cdc5 and Clb-Cdk, the kinases responsible for Cfi/Net1 phosphorylation, and PP2A^Cdc55^, the phosphatase complex that counteracts this phosphorylation. Recently, Zds1 and Zds2, previously identified to associate with the chromatin silencing machinery [56], have been shown to play a role in early anaphase release of Cdc14 and to interact with PP2A^Cdc55^ [20], suggesting another potential mediator for the histone modification requirement for efficient mitotic exit.

As all of the histone modifications studied here are conserved from yeast to mammals, it seems possible that their role in controlling localization of Cdc14 is also conserved. A hint that this might be the case comes from studies of HUB1, the homolog of Bre1 in *Arabidopsis thaliana*. Cells from *hub1-1* mutants exhibit an increased population with 4C DNA content, consistent with a delay in progression through G2 or mitosis [57]. In addition, while Cdc14 is not essential for mitotic exit in animal cells, its cell cycle-regulated association with the nucleolus is remarkably conserved from yeast to humans [58]. Recently, it was reported that mammalian cells have adapted the Cdc14 sequestration mechanism to regulate the G2 DNA-damage checkpoint [59]. Given the emerging role of meH3K79 and other histone modifications in the DNA damage response [60], it is possible that the Cdc14 regulation by meH3K79 described here is relevant to the function in metazoans of this conserved histone modification in DNA damage checkpoint signaling.

More generally, our studies point to a role for histone modifications in a process involving chromatin but not directly impinging on transcription or DNA repair on which most functional studies have been focused. The nonredundant role for all three known histone methylations in mitotic exit suggests they do not function in this context as part of a combinatorial code but instead may affect chromatin in a similar way to promote the programmed release of a chromatin bound factor.

## Materials and Methods

### Yeast Strains and Plasmids

A list of the yeast strains and plasmids used in this paper is found in Table S1. Knockout strains were generated by homologous recombination by transforming the parent strain with PCR products encoding drug or auxotrophic markers flanked by targeting homology.

### Measurement of Genetic Interactions

Genetic interactions of mutants with *lte1Δ* mutations were assayed by sporulation and tetrad dissection of heterozygous diploids containing a wild type copy of *LTE1* on the *CEN URA3* pRS316 plasmid (pRS316-*LTE1*). Spores with the respective single, double, and triple mutations and the pRS316-*LTE1* plasmid were selected for and grown overnight on YPD plates to increase the population of cells that have lost the plasmid. To assay for growth of mutants without *LTE1*, strains were inoculated from frozen stocks into minimal synthetic defined liquid media lacking uracil (SD –Ura) and grown overnight to select for pRS316-*LTE1*, and diluted into liquid YPD media and grown overnight to allow for loss of the plasmid. Five-fold dilutions of 1 OD of cells were made in water and transferred onto plates containing the drug 5-fluoroorotic acid (5-FOA) to select against cells that still harbor the *URA3*-marked plasmid or SD –Ura plates to control for amount of cells plated. The wild type and *lte1Δ* mutants shown for Figure 2, Figure 4, and Figure 5 are from tetrad dissections of YM2589 and are representative of the growth of wild type and *lte1Δ* mutants from tetrad dissection of YM3477, YM3463, YM2924, YM3482, YM3476, and YM3447.

### Cell Cycle Time Course for Cdc14 Localization

*cdc15-2* strains were inoculated from frozen stocks and grown overnight at 25°C in YPD medium and then diluted and grown an additional night until saturation. Cultures were diluted into 25 ml of YPD at room temperature and grown for at least one doubling until OD 0.2 at 25°C. Alpha-factor (25 µl of 10 mg/ml) was added twice to each culture for a total of 2.5 hrs to arrest cells in G1. To release cells into the cell cycle, cells were first collected on nitrocellulose filters and washed with 5×25 ml of YPD to eliminate residual alpha factor. Cells were then resuspended in 25 mL of YPD pre-warmed to 37°C at the 0 min timepoint to inactivate the *cdc15-2* gene product and incubated until time course completion. 1 ml samples were collected at each timepoint and processed as described below. Time courses with CDC15 strains were performed as above except cells were resuspended at timepoint 0 min in 25°C YPD media and incubated at 25°C during the time course.

### Indirect Immunofluorescence

Samples collected from time course are resuspended in 1 ml Buffer KP+formaldehyde (90 mM potassium phosphate pH 6.4, 3.7% formaldehyde) and split into two 500 µl fractions. Fraction #1 (for Cdc14-HA localization) is fixed by incubation at 30°C for 10 min (mixed on BD Clay Adams Nutator). Fraction #2 (for spindle visualization) is fixed overnight at 4°C without mixing (overnight fixation increases spindle signal). Both samples are washed 3× after fixation with 1 ml of Buffer KP (100 mM potassium phosphate pH 6.4) and once with 1 ml Buffer KPS (100 mM K_2_HPO_4_, 1.2 M sorbitol, 36 mM citric acid, pH 5.9). Samples are resuspended in 250 µl of Buffer KPS and stored overnight at −20°C. Samples are defrosted and digested by addition of 2 µl of 10 mg/ml Zymolyase 100T (#120493, Seikagaku America) and 20 µl of Glusulase (#NEE154001EA, PerkinElmer) at 30°C with nutation (Fraction #1 samples digested 15 min while Fraction #2 samples digested 30 min). Digested cells are washed once with Buffer KPS (after digestion spin at 2000 rpm) and resuspended in ∼20–40 µl Buffer KPS depending on size of pellet. 10 µl of cells are applied for 2 min to glass slides with wells (ER-267W, Erie Scientific) that have been pre-coated with 0.1% poly-L-lysine for 5 min. Cells are aspirated and slides are plunged sequentially into cold methanol for 3 min and 10 sec into cold acetone (methanol and acetone were stored at −20°C). Wells were washed twice with BSA-PBS (1× PBS with 10 mg/ml BSA) and incubated with 20 µl of either anti-HA (MMS-101R, Covance, diluted 1∶150) or anti-tubulin (NB100-1639, Novus, diluted 1∶250) antibody in BSA-PBS for 1.5 hrs in a humid chamber. Wells were washed 5× with BSA-PBS and incubated with 20 µl of either anti-mouse TRITC conjugated secondary or anti-rat FITC conjugated secondary antibody (715-025-150, 712-095-150, Jackson ImmunoReseach, both diluted 1∶1000) in BSA-PBS for 2 hrs in a humid chamber. Wells are washed 5× with BSA-PBS and mounted in mounting media (1 mg/mL phenylenediamine, 40 mM K_2_HPO, 10 mM KH_2_PO_4_, 150 mM NaCl, 0.1% NaN_3_, 50 µg/mL DAPI, 5% vectashield, 90% glycerol, pH 8). Slides are analyzed with the Zeiss Axiovert 200 M inverted microscope and images were analyzed with AxioVision 4.6 software. For spindle measurements, slides were analyzed with the DeltaVision Spectris deconvolution microscope and images were analyzed using the softWoRx software suite.

### Reverse Transcription Coupled to Quantitative PCR Analysis (RT–QPCR)

Saturated overnight cultures are diluted to OD 0.05 in 25 mL of YPD and grown at 30°C until OD 0.4–0.5. Cells are washed with ice-cold water and snap-frozen. Pellets are resuspended in 1 ml of TRIzol (15596-026, Invitrogen) and zirconia/silica beads were added. Cells were disrupted in a Mini-Beadbeater-8 by two cycles of disruption at full power for 2.5 min, cooling on ice for 2.5 min between each cycle. Lysates were clarified by centrifugation (13,500 rpm for 10 min at 4°C) and transferred to a fresh tube. 200 µl chloroform was added, vortexed, incubated for 10 min at room temperature, and spun at 13,500 rpm for 10 min at 4°C. Aqueous phase was treated with 500 µl chloroform as above. 500 µl isopropanol was added to the aqueous phase, incubated for 15 min at 4°C, and spun at 13,500 rpm for 10 min at 4°C. Pellets are washed with 1 ml 75% ethanol, dried by speed vac, resuspended in 100 µl water, and incubated at 55°C for 1 hr. 40 µg of total RNA was diluted in 72 µl of water per replicate (each RNA sample was treated in triplicate) and treated with TURBO DNA-free kit (1907, Ambion). Approximately 15 µg of total RNA was used for each RT and minus RT 50 µl reaction (50 mM Tris-HCl pH 8.4, 75 mM KCl, 0.5 µg/µl dT_20_N oligonucleotide). Reactions were incubated for 10 min at 70°C and 10 min on ice. 50 µl enzyme/dNTP mix (50 mM Tris-HCl pH 8.4, 75 mM KCl, 6 mM MgCl2, 20 mM DTT, 1 mM dNTP, 2× RT) was added to the reactions and were incubated overnight at 42°C. Samples were treated with 2 µl of 0.5 M EDTA, vortexed, 10 µl of 1 N NaOH was added and incubated for 10 min at 65°C, and subsequently neutralized with 10 µl of 1 N HCl. cDNA was purified using Zymo DNA Clean & Concentrator columns (D4004) and samples were diluted 1∶100 for qPCR analysis. Sequences of primers used available upon request.

### Western Blotting

This protocol was modified from [61]. Cells (6 OD) are collected at ∼0.7–0.8 OD after growth for at least two doublings. Cell pellets were washed with ice-cold water and flash-frozen with liquid N_2_ and stored at −80°C. Frozen cell pellets were resuspended in 240 µl of Sample Buffer (50 mM Tris pH 7.5, 5 mM EDTA, 5% SDS, 10% glycerol, 0.5% 2-mercaptoethanol, 1∶100 dilution of Phosphatase Inhibitor Cocktail 1 and 2 [P2850 and P5726, Sigma], 1∶260 dilution of Protease Inhibitor Cocktail [P8215, Sigma], 100 µM PMSF, bromophenol blue for color) preheated to 100°C and incubated in 100°C heat block for 3–5 min. Approximately 100 µl of zirconia/silica beads were added to each tube and cells were lysed by beadbeating at full power with Mini-Beadbeater-8 (#693, Bio Spec) for 3 min. Tubes are centrifuged briefly and incubated at 100°C for 3 min, vortexed, and centrifuged at 14K rpm for 10 min. 10 µl of supernatant is loaded and run on 4–15% Tris-HCI SDS-PAGE gel (#3450029, Bio-Rad) and transferred to nitrocellulose for 16 hr (25 V at 4°C) in SDS transfer buffer (20 mM Tris base, 150 mM glycine, 0.375% SDS, 20% methanol). Blots are cut below a 28 kDa marker in order to simultaneously probe for different primaries and are blocked for 30 min with TBST (10 mM Tris-HCl pH 8.0, 150 mM NaCl, 0.05% Tween 20)+5% milk, incubated with either anti-HA (diluted 1∶1500), anti-myc 9E10 (sc-40, Santa Cruz, diluted 1∶2000), or anti-dimeH3K4 (#07-030, Upstate, diluted 1∶1000) in TBST+5% milk for 1 hr, washed 5× for 5 min with TBST, incubated with either anti-mouse or rabbit HRP secondary (170-6515, 170-6516, Bio-Rad, diluted 1∶5000) in TBST+5% milk for 30 min, and washed with 5× for 5 min with TBST. Blots are visualized by enhanced chemiluminescence substrate (#34080, Thermo Scientific) and are stripped and reprobed by anti-Pgk1 antibody to control for loading (A6457, Molecular Probes, diluted 1∶10000).

### Chromatin Immunoprecipitation (ChIP)

Cells were grown to mid-log phase (OD ∼0.4). 50 ml of cells were fixed with 1% formaldehyde at room temperature for 15 min. Glycine was added to a final concentration of 125 mM and samples were incubated at room temperature for 5 min. Cells were washed twice with TBS (20 mM Tris-HCl pH 7.6, 150 mM NaCl), collected by centrifugation and snap-frozen. Pellets were resuspended in 500 µl of lysis buffer (50 mM HEPES pH 7.5, 140 mM NaCl, 1 mM EDTA, 1% Triton X-100, 0.1% sodium deoxycholate) with 1∶260 Sigma Protease Inhibitor Cocktail) and zirconia/silica beads were added. Cells were disrupted in a Mini-Beadbeater-8 by five cycles of disruption at full power for 1 min, cooling on ice for 2 min between each cycle. The lysate was collected. The pellet containing chromatin was separated by centrifugation and resuspended in 300 µl lysis buffer. Sonication was performed using a waterbath sonicator (Bioruptor) set to cycle at 30 sec on and 1 min off for 30 min. Cell debris was removed by centrifugation at 14,000 rpm for 10 min. 1 µl of anti-HA antibody was added to each sample and the mixture was nutated at 4°C for 2 hr. Protein G Sepharose (#17-0618-01, GE Healthcare) was washed 3× in PBS and 2× in lysis buffer. 20 µl of washed beads were added to each sample. After 1.5 hr at 4°C the beads were washed 2× with lysis buffer, 2× with high salt lysis buffer (50 mM HEPES pH 7.5, 500 mM NaCl, 1 mM EDTA, 1% Triton X-100, 0.1% sodium deoxycholate), 2× with wash buffer (10 mM Tris-HCl pH 8.0, 250 mM LiCl, 0.5% NP-40, 0.5% sodium deoxycholate, 1 mM EDTA) and once with TE pH 8.0. 100 µl of elution buffer (50 mM Tris-HCl pH 8.0, 10 mM EDTA, 1% SDS) was added and the samples were incubated at 65°C for 15 min. The eluate was collected by centrifugation. The beads were washed with TE+0.67% SDS+proteinase K and the supernatant was added to the eluate from the previous step and incubated at 65°C overnight. DNA was purified using Qiagen PCR purification columns. RNAse A was added to the purified DNA and the samples were incubated at 37°C for 1 hr. Samples were subjected to quantitative PCR analysis (primers used were from [62]).

### FACS Analysis

2.5 ml cells were collected in 10 min intervals after alpha-factor arrest as described above. Cells were pelleted and resuspended in 5 ml of 70% ethanol to fix overnight at 4°C. Samples are washed twice in 750 µl of 50 mM Tris-HCl pH 8 with 5 mM EDTA. Cells are resuspended in 500 µl of RNase A solution (2 mg/ml RNase [R5503, Sigma], 50 mM Tris-Cl pH 8, 5 mM EDTA previously boiled for 15 min and cooled to room temperature on the bench) and incubated for 4 hours at 37°C. Cells are pelleted, resuspended in 200 µl of Pepsin solution (5 mg/ml Pepsin [P7000, Sigma] in 55 mM HCl), and incubated for 20 min at 37°C. Samples are washed once in 750 µl of 50 mM Tris-HCl pH 7.5 with 5 mM EDTA and resuspended in 500 µl of the same buffer. 50 µl of cells added to 1 mL of Sytox solution (1 µM Sytox Green [S7020, Molecular Probes], 50 mM Tris-HCl pH 7.5, 5 mM EDTA) and are sonicated to break up cell clumps (Branson 450 Sonifier). Samples are incubated overnight at 4°C and analyzed the next day by flow cytometry with the BD FACSCalibur system.

## Supporting Information

Figure S1 Synthetic lethal interactions between *bre1Δ*, *rad6Δ*, and *lge1Δ* can be suppressed by deletion of *BUB2*. Diploid strains heterozygous for *lte1Δ*, *bub2Δ*, and either the *bre1Δ*, *rad6Δ* or *lge1Δ* mutations are sporulated and analyzed by tetrad dissection. The red circles refer to spores that are predicted by the genotypes of the other spores in the tetrad to be double mutants of *lte1Δ* with *bre1Δ*, *rad6Δ*, or *lge1Δ*. The blue circles refer to spores that are triple mutants for *lte1Δ* and *bub2Δ* in combination with *bre1Δ*, *rad6Δ*, and *lge1Δ*. Spores that are missing without a red circle are predicted to be *lte1Δ* single mutants, which appear to have sporadic viability or germination issues.
(8.89 MB TIF)

Figure S2 Cdc14 release profile of *cdc15-2 mad1Δ* and *cdc15-2 mad1Δ bre1Δ* mutants by measuring spindle length. The profile of Cdc14 release from nucleolar sequestration is displayed for *cdc15-2 mad1Δ and cdc15-2 mad1Δ bre1Δ* mutants as a function of the spindle length of each individual cell. Alpha-factor arrested cells are released into YPD media at 37°C to inactivate the *cdc15-2* mutant gene product and the 90 minute (for *cdc15-2 mad1Δ*) and 110 minute (for *cdc15-2 mad1Δ bre1Δ*) timepoints are processed for indirect immunofluorescence as described in Figure 2B. Approximately 125–150 cells were quantified for each strain for spindle length measurements and for full or partial release of Cdc14-HA.
(0.52 MB TIF)

Figure S3 Cell cycle progression of *cdc15-2 mad1Δ* and *cdc15-2 mad1Δ bre1Δ* mutants. The DNA content profile of *cdc15-2 mad1Δ* and *cdc15-2 mad1Δ bre1Δ* mutants released from alpha-factor arrest are measured by flow cytometry. Alpha-factor arrested cells are released into YPD media at 37°C to inactivate *the cdc15-2* mutant gene product. Samples taken at 10 minute intervals are fixed with 70% ethanol. 20,000 cells were measured for each timepoint for 1C or 2C DNA content by measurement of fluorescence from SYTOX incorporation. Profiles labeled in blue are timepoints that are undergoing transition from G1 to G2 content.
(0.93 MB TIF)

Table S1 Strains and plasmids.
(0.10 MB DOC)
