# Supplementary figures and images for: Nonredundant Requirement for Multiple Histone Modifications for the Early Anaphase Release of the Mitotic Exit Regulator Cdc14 from Nucleolar Chromatin
Source: PLoS Genet. 2009 Aug 7;5(8):e1000588. doi: 10.1371/journal.pgen.1000588 (PMC2716543; doi:10.1371/journal.pgen.1000588)

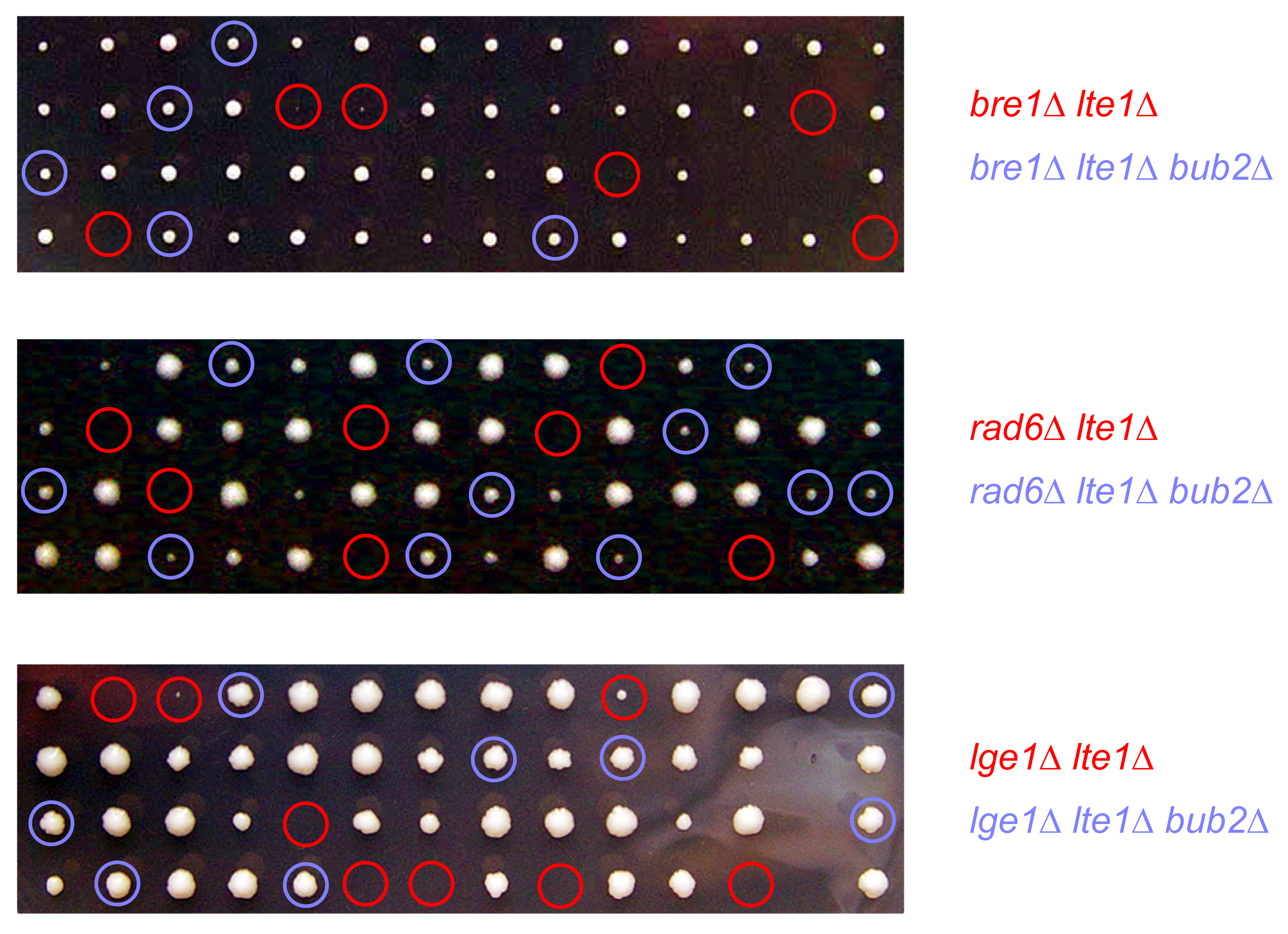

Supplement: Figure S1 — Synthetic lethal interactions between bre1Δ, rad6Δ, and lge1Δ can be suppressed by deletion of BUB2. Diploid strains heterozygous for lte1Δ, bub2Δ, and either the bre1Δ, rad6Δ or lge1Δ mutations are sporulated and analyzed by tetrad dissection. The red circles refer to spores that are predicted by the genotypes of the other spores in the tetrad to be double mutants of lte1Δ with bre1Δ, rad6Δ, or lge1Δ. The blue circles refer to spores that are triple mutants for lte1Δ and bub2Δ in combination with bre1Δ, rad6Δ, and lge1Δ. Spores that are missing without a red circle are predicted to be lte1Δ single mutants, which appear to have sporadic viability or germination issues. (8.89 MB TIF) [file pgen.1000588.s001.tif]

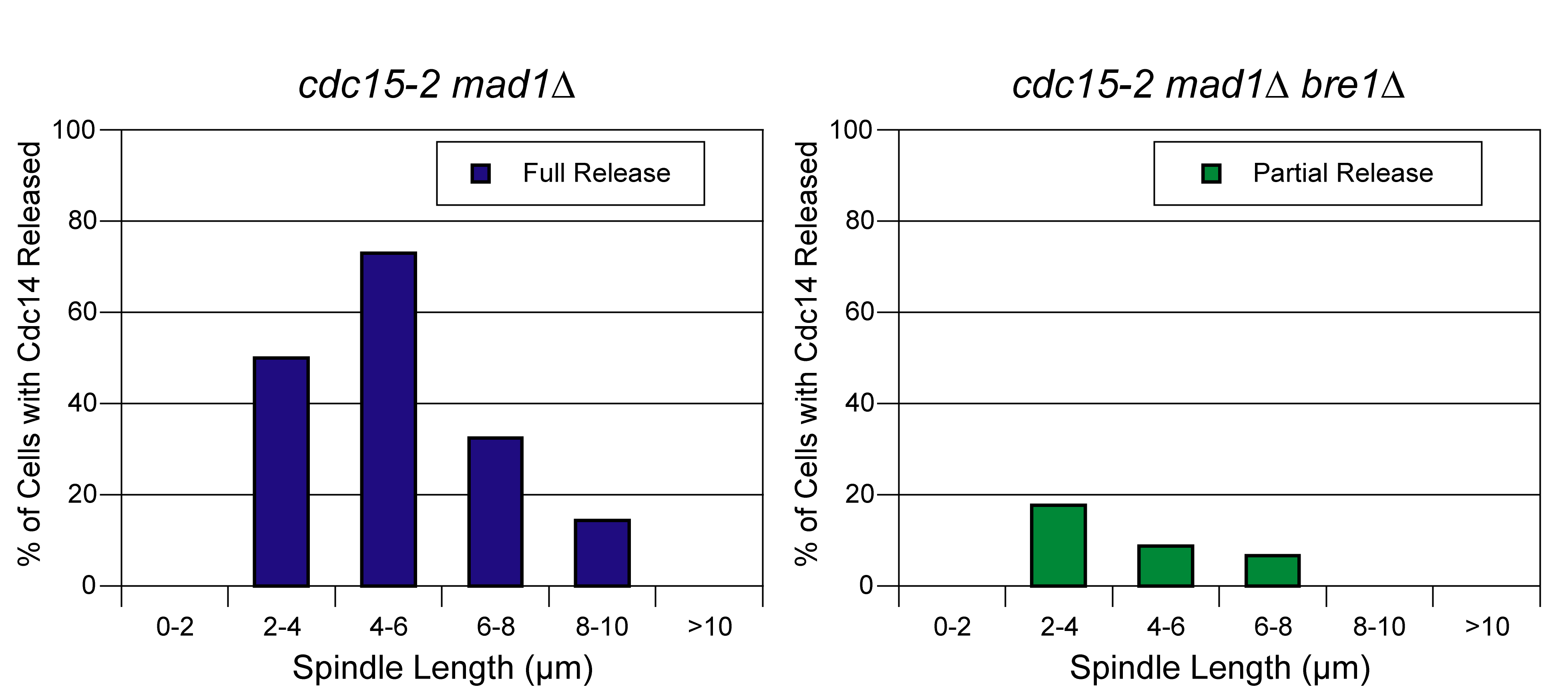

Supplement: Figure S2 — Cdc14 release profile of cdc15-2 mad1Δ and cdc15-2 mad1Δ bre1Δ mutants by measuring spindle length. The profile of Cdc14 release from nucleolar sequestration is displayed for cdc15-2 mad1Δ and cdc15-2 mad1Δ bre1Δ mutants as a function of the spindle length of each individual cell. Alpha-factor arrested cells are released into YPD media at 37°C to inactivate the cdc15-2 mutant gene product and the 90 minute (for cdc15-2 mad1Δ) and 110 minute (for cdc15-2 mad1Δ bre1Δ) timepoints are processed for indirect immunofluorescence as described in Figure 2B. Approximately 125–150 cells were quantified for each strain for spindle length measurements and for full or partial release of Cdc14-HA. (0.52 MB TIF) [file pgen.1000588.s002.tif]

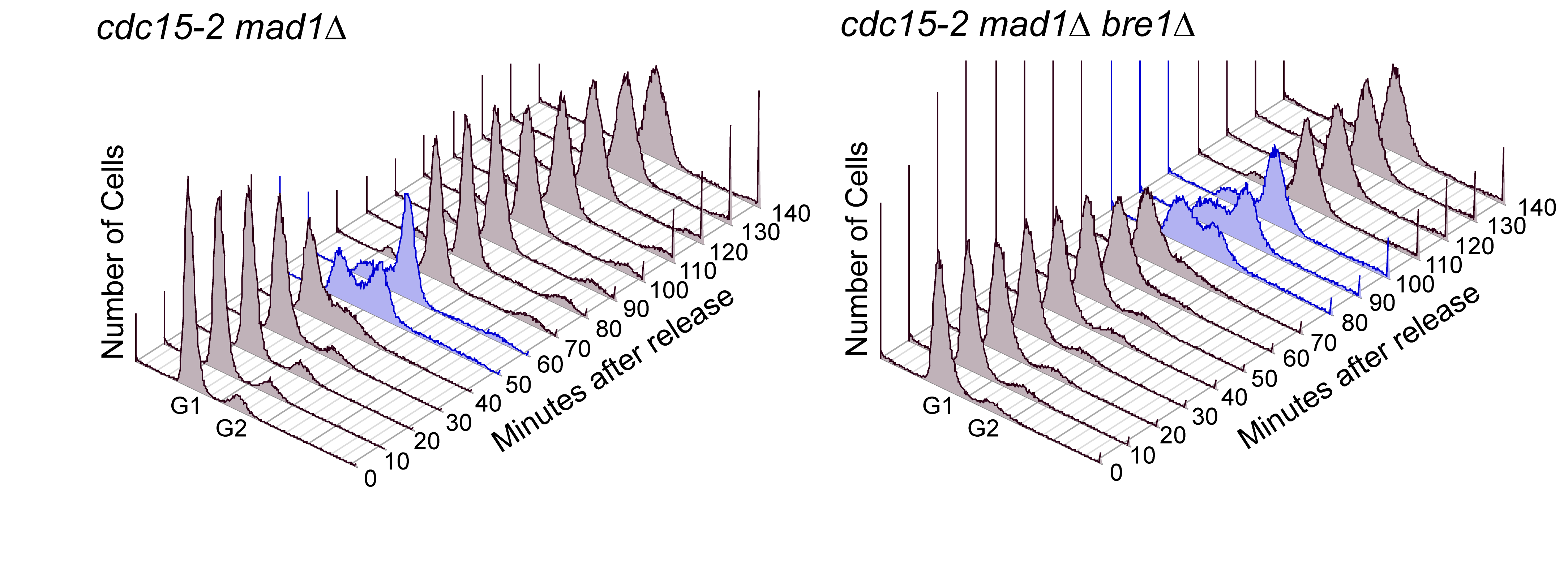

Supplement: Figure S3 — Cell cycle progression of cdc15-2 mad1Δ and cdc15-2 mad1Δ bre1Δ mutants. The DNA content profile of cdc15-2 mad1Δ and cdc15-2 mad1Δ bre1Δ mutants released from alpha-factor arrest are measured by flow cytometry. Alpha-factor arrested cells are released into YPD media at 37°C to inactivate the cdc15-2 mutant gene product. Samples taken at 10 minute intervals are fixed with 70% ethanol. 20,000 cells were measured for each timepoint for 1C or 2C DNA content by measurement of fluorescence from SYTOX incorporation. Profiles labeled in blue are timepoints that are undergoing transition from G1 to G2 content. (0.93 MB TIF) [file pgen.1000588.s003.tif]
