# Supplementary material for: Nonredundant Requirement for Multiple Histone Modifications for the Early Anaphase Release of the Mitotic Exit Regulator Cdc14 from Nucleolar Chromatin
Source: PLoS Genet. 2009 Aug 7;5(8):e1000588. doi: 10.1371/journal.pgen.1000588 (PMC2716543; doi:10.1371/journal.pgen.1000588)
Supplement: Table S1 — Strains and plasmids. (0.10 MB DOC) [file pgen.1000588.s004.doc]

| **Table S1 - List of strains used in this study** | |  |
| --- | --- | --- |
| **Strain #** | **Genotype** | **Source** |
| A2853 | MAT**a***, ade2-1 leu2-3 ura3 trp1-1 his3-11,15 can1-100 mad1::URA3 CDC14-3HA* | A. Amon [16] |
| A4300 | A2853 *cdc15-2* | A. Amon [16] |
| A4302 | A2853 *slk19∆::KanMX6* | A. Amon [16] |
| A4304 | A4300 *slk19∆::KanMX6* | A. Amon [16] |
| YM1740 | MAT***a*** *his3∆1 leu2∆0 ura3∆0 met15∆0 bre1∆::KanMX4* | Res. Genetics |
| YM1768 | MAT***a*** *his3∆1 leu2∆0 ura3∆0 met15∆0 lys2∆0* | This study |
| YM2587 | MAT**a/α** *his3∆1/his3∆1 leu2∆0/leu2∆0 ura3∆0/ura3∆0 met15∆0/MET15*  *lys2∆0/LYS2 lte1∆::HygMX4/LTE1 bre1∆::KanMX4/BRE1 bub2∆::NatMX4/BUB2* | This study |
| YM2588 | MAT**a/α** *his3∆1/his3∆1 leu2∆0/leu2∆0 ura3∆0/ura3∆0 met15∆0/MET15 lys2∆0/LYS2 lte1∆::HygMX4/LTE1 rad6∆::KanMX4/RAD6 bub2∆::NatMX4/BUB2* | This study |
| YM2589 | MAT**a/α** *his3∆1/his3∆1 leu2∆0/leu2∆0 ura3∆0/ura3∆0 met15∆0/MET15 lys2∆0/LYS2 lte1∆::HygMX4/LTE1 bre1∆::KanMX4/BRE1 bub2∆::NatMX4/BUB2 + pRS316-LTE1* | This study |
| YM2590 | MAT**a/α** *his3∆1/his3∆1 leu2∆0/leu2∆0 ura3∆0/ura3∆0 met15∆0/MET15 lys2∆0/LYS2 lte1∆::HygMX4/LTE1 lge1∆::KanMX4/LGE1 bub2∆::NatMX4/BUB2* | This study |
| YM2601 | A2853 *bre1∆::NatMX4* | This study |
| YM2602 | A4300 *bre1∆::NatMX4* | This study |
| YM2858 | MAT**a***, ade2-1 leu2-3 ura3 trp1-1 his3-11,15 can1-100 SLK19-13myc::KanMX6* | This study |
| YM2924 | MAT**a/α** *his3∆1/his3∆1 leu2∆0/leu2∆0 ura3∆0/ura3∆0 met15∆0/MET15 lys2∆0/lys2∆0 lte1∆::HygMX4/LTE1 htb1-K123R::NatMX4/HTB1 htb2-K123R::HISMX6/HTB2 + pRS316-LTE1* | This study |
| YM2927 | A4300 *htb1-K123R::NatMX4 htb2-K123R::HISMX6* | This study |
| YM3144 | A4300 *dot1∆::NatMX4* | This study |
| YM3145 | A4300 *set1∆::NatMX4* | This study |
| YM3179 | MAT**a***, ade2-1 leu2-3 ura3 trp1-1 his3-11,15 can1-100 NET1-3HA::KanMX6* | This study |
| YM3180 | MAT**a***, ade2-1 leu2-3 ura3 trp1-1 his3-11,15 can1-100 FOB1-3HA::KanMX6* | This study |
| YM3205 | YM3179 *bre1∆::NatMX4* | This study |
| YM3206 | YM3180 *bre1∆::NatMX4* | This study |
| YM3208 | MAT**a***, ade2-1 leu2-3 ura3 trp1-1 his3-11,15 can1-100 SPO12-13myc::KanMX6* | This study |
| YM3209 | YM3208 *bre1∆::NatMX4* | This study |
| YM3316 | A4300 *spo12∆::NatMX4 bns1∆::HygMX4* | This study |
| YM3422 | A4300 *sir2∆::HygMX4* | This study |
| YM3423 | YM2927 *sir2∆::HygMX4* | This study |
| YM3424 | A4304 *sir2∆::HygMX4* | This study |
| YM3427 | YM3316 *sir2∆::KanMX6* | This study |
| YM3446 | A4300 *set2∆::NatMX4* | This study |
| YM3447 | MAT**a/α** *his3∆1/his3∆1 leu2∆0/leu2∆0 ura3∆0/ura3∆0 met15∆0/MET15 lys2∆0/lys2∆0 lte1∆::HygMX4/LTE1 rpd3::NatMX4/RPD3 + pRS316-LTE1* | This study |
| YM3461 | A4300 *rpd31∆::NatMX4* | This study |
| YM3462 | A4300 *hda1∆::NatMX4* | This study |
| YM3463 | MAT**a/α** *his3∆1/his3∆1 leu2∆0/leu2∆0 ura3∆0/ura3∆0 met15∆0/MET15 lys2∆0/lys2∆0 lte1∆::HygMX4/LTE1 hda1::NatMX4/HDA1 + pRS316-LTE1* | This study |
| YM3476 | MAT**a/α** *his3∆1/his3∆1 leu2∆0/leu2∆0 ura3∆0/ura3∆0 met15∆0/MET15 lys2∆0/LYS2 set1∆::HisMX4/SET1 set2∆::KanMX4/SET2 lte1∆::NatMX4/LTE1 + pRS316-LTE1* | This study |
| YM3477 | MAT**a/α** *his3∆1/his3∆1 leu2∆0/leu2∆0 ura3∆0/ura3∆0 met15∆0/MET15 lys2∆0/LYS2 set1∆::HisMX4/SET1 dot1∆::KanMX4/DOT1 lte1∆::NatMX4/LTE1 + pRS316-LTE1* | This study |
| YM3482 | MAT**a/α** *his3∆1/his3∆1 leu2∆0/leu2∆0 ura3∆0/ura3∆0 met15∆0/MET15 lys2∆0/LYS2 lte1∆::HygMX4/LTE1 bre1∆::KanMX4/BRE1 sir2∆::NatMX4/SIR2 + pRS316-LTE1* | This study |
| YM3484 | YM2858 *bre1∆::NatMX4* | This study |
| YM3486 | MAT**a***, ade2-1 leu2-3,112 ura3-1 trp1-1 his3-11 can1-100 bar::hisG CDC15-3HA* | D. Morgan |
| YM3487 | MAT**a***, ade2-1 leu2-3 ura3 trp1-1 his3-11,15 can1-100 CDC55-3HA::KanMX6* | This study |
| YM3489 | MAT**a***, ade2-1 leu2-3 ura3 trp1-1 his3-11,15 can1-100 TPD3-3HA::KanMX6* | This study |
| YM3494 | YM3487 *bre1∆::NatMX4* | This study |
| YM3496 | YM3489 *bre1∆::NatMX4* | This study |
| YM3498 | YM3486 *bre1∆::NatMX4* | This study |
| YM3500 | MAT**a** *his3∆1 leu2∆0 ura3∆0 MET15 LYS2 lte1∆::HygMX4 bre1∆::KanMX4 + pRS316-LTE1* | This study |
| YM3501 | MAT**a** *his3∆1 leu2∆0 ura3∆0 MET15 lys2∆0 bre1∆::KanMX4 + pRS316-LTE1* | This study |
| YM3502 | MAT**a** *his3∆1 leu2∆0 ura3∆0 MET15 lys2∆0 lte1∆::HygMX4 + pRS316-LTE1* | This study |
| YM3503 | MAT**a** *his3∆1 leu2∆0 ura3∆0 met15∆0 lys2∆0 + pRS316-LTE1* | This study |
| YM3504 | MAT**a** *his3∆1 leu2∆0 ura3∆0 MET15 lys2∆0 lte1∆::HygMX4 htb1-K123R::NatMX4 htb2-K123R::HISMX6 + pRS316-LTE1* | This study |
| YM3505 | MAT**a** *his3∆1 leu2∆0 ura3∆0 met15∆0 lys2∆0 htb1-K123R::NatMX4 htb2-K123R::HISMX6 + pRS316-LTE1* | This study |
| YM3506 | MAT**a** *his3∆1 leu2∆0 ura3∆0 met15∆0 LYS2 dot1∆::KanMX4 lte1∆::NatMX4 set1∆::HISMX6 + pRS316-LTE1* | This study |
| YM3507 | MAT**a** *his3∆1 leu2∆0 ura3∆0 met15∆0 LYS2 dot1∆::KanMX4 set1∆::HISMX6 + pRS316-LTE1* | This study |
| YM3508 | MAT**a** *his3∆1 leu2∆0 ura3∆0 met15∆0 LYS2 lte1∆::NatMX4 set1∆::HISMX6 + pRS316-LTE1* | This study |
| YM3509 | MAT**a** *his3∆1 leu2∆0 ura3∆0 MET15 lys2∆0 dot1∆::KanMX4 lte1∆::NatMX4 + pRS316-LTE1* | This study |
| YM3510 | MAT**a** *his3∆1 leu2∆0 ura3∆0 MET15 lys2∆0 set1∆::HISMX6 + pRS316-LTE1* | This study |
| YM3511 | MAT**a** *his3∆1 leu2∆0 ura3∆0 met15∆0 LYS2 dot1∆::KanMX4 + pRS316-LTE1* | This study |
| YM3513 | MAT**?** *his3∆1 leu2∆0 ura3∆0 met15∆0 lys2∆0 sir2∆::NatMX4 lte1∆::HygMX4 + pRS316-LTE1* | This study |
| YM3514 | MAT**?** *his3∆1 leu2∆0 ura3∆0 met15∆0 lys2∆0 sir2∆::NatMX4 + pRS316-LTE1* | This study |
| YM3515 | MAT**a** *his3∆1 leu2∆0 ura3∆0 MET15 LYS2 set2∆::KanMX4 lte1∆::NatMX4 set1∆::HISMX6 + pRS316-LTE1* | This study |
| YM3516 | MAT**a** *his3∆1 leu2∆0 ura3∆0 met15∆0 lys2∆0 set2∆::KanMX4 set1∆::HISMX6 + pRS316-LTE1* | This study |
| YM3517 | MAT**a** *his3∆1 leu2∆0 ura3∆0 MET15 lys2∆0 lte1∆::NatMX4 set1∆::HISMX6 + pRS316-LTE1* | This study |
| YM3518 | MAT**a** *his3∆1 leu2∆0 ura3∆0 MET15 lys2∆0 set2∆::KanMX4 lte1∆::NatMX4 + pRS316-LTE1* | This study |
| YM3519 | MAT**a** *his3∆1 leu2∆0 ura3∆0 MET15 LYS2 set2∆::KanMX4 + pRS316-LTE1* | This study |
| YM3520 | MAT**a** *his3∆1 leu2∆0 ura3∆0 MET15 lys2∆0 lte1∆::HygMX4 rpd3∆::NatMX4 + pRS316-LTE1* | This study |
| YM3521 | MAT**a** *his3∆1 leu2∆0 ura3∆0 MET15 lys2∆0 rpd3∆::NatMX4 + pRS316-LTE1* | This study |
| YM3522 | MAT**a** *his3∆1 leu2∆0 ura3∆0 MET15 lys2∆0 lte1∆::HygMX4 hda1∆::NatMX4 + pRS316-LTE1* | This study |
| YM3523 | MAT**a** *his3∆1 leu2∆0 ura3∆0 met15∆0 lys2∆0 hda1∆::NatMX4 + pRS316-LTE1* | This study |
| YM3682 | MAT**a***, ade2-1 leu2-3 ura3 trp1-1 his3-11,15 can1-100 mad1::URA3 CDC14-3HA hht1-hht1∆::NatMX4 hht2-hht2∆::TRP cdc15-2::KanMX6 + pRS415-HHT2-HHF2)* | This study |
| YM3683 | MAT**a***, ade2-1 leu2-3 ura3 trp1-1 his3-11,15 can1-100 mad1::URA3 CDC14-3HA hht1-hht1∆::NatMX4 hht2-hht2∆::TRP cdc15-2::KanMX6 + pRS415-hht2-K4R-HHF2)* | This study |
| YM3684 | MAT**a***, ade2-1 leu2-3 ura3 trp1-1 his3-11,15 can1-100 mad1::URA3 CDC14-3HA hht1-hht1∆::NatMX4 hht2-hht2∆::TRP cdc15-2::KanMX6 + pRS415-hht2-K79R-HHF2)* | This study |
| YM3685 | MAT**a***, ade2-1 leu2-3 ura3 trp1-1 his3-11,15 can1-100 mad1::URA3 CDC14-3HA hht1-hht1∆::NatMX4 hht2-hht2∆::TRP cdc15-2::KanMX6 + pRS415-hht2-K36R-HHF2)* | This study |
